# Prognostic value of α2δ1 in hypopharyngeal carcinoma: A retrospective study

**DOI:** 10.1515/med-2021-0356

**Published:** 2021-09-16

**Authors:** Qiang Liu, Yanbo Dong, Shuoqing Yuan, Minghang Yu, Liangfa Liu, Qing Zhang

**Affiliations:** Department of Oncology, Beijing Hospital of Traditional Chinese Medicine, Capital Medical University, Beijing 100010, China; Department of Otolaryngology Head and Neck Surgery, Beijing Friendship Hospital, Capital Medical University, Beijing 100050, China; Department of Immunology, School of Basic Medical Sciences, Capital Medical University, Beijing 100069, China

**Keywords:** hypopharyngeal carcinoma, α2δ1, immunohistochemistry, survival analysis, prognosis

## Abstract

Voltage-dependent calcium channel subunit alpha-2/delta-1 (α2δ1) has been identified as a marker of cancer stem cells in multiple malignant tumor types. However, α2δ1’s role in the prognosis of hypopharyngeal squamous cell carcinoma (HSCC) was not reported. In our study, ten pairs of HSCC and peritumoral normal tissues were used for immunohistochemistry assessment. And α2δ1 expression levels of 34 more HSCC samples were also evaluated, represented by the integral optic density using Image-Pro Plus. Clinicopathological associations and prognostic value of α2δ1 were analyzed. As a result, α2δ1 expression was frequently increased in HSCC tissues. Although the correlation between patients’ clinicopathological characteristics and their α2δ1 expression levels was not significant, α2δ1 expression was significantly correlated with unfavorable overall survival (OS) (*P* = 0.018) and progression-free survival (PFS) (*P* = 0.023). Univariate and multivariate cox regression analyses suggested α2δ1’s prognostic role for both OS and PFS (*P* = 0.013 and 0.011, respectively). This study specifically demonstrated that α2δ1 regularly increased in HSCC compared with peritumoral tissues, and α2δ1 could act as a promising prognostic marker in HSCC patients.

## 1 Introduction

Hypopharyngeal squamous cell carcinoma (HSCC), as a subtype of head and neck cancer, is not very common, with an incidence of less than ten cases/million people each year [1,2]. However, it is one of the most life-threatening types of head and neck cancer, with high malignancy, poor prognosis, and high morbidity [3]. The disease stands out by the undermentioned aspects: intricate anatomy of the primary tumor sites and thus difficulty in early diagnosis; tendency of local recurrence, metastasis, and secondary primary tumors. So far clinical and experimental exploration has not enhanced the 5-year overall survival (OS) rate of HSCC to a satisfactory level [4]. Identifying prognostic markers could be helpful in improving prognosis and guiding personalized treatments.

Voltage-dependent calcium channel subunit alpha-2/delta-1 (α2δ1), encoded by CACNA2D1 gene, is a member of the α2/δ subunit family. α2/δ subunit is reported as auxiliary subunits of calcium channels located at the plasma membrane, whose function is to increase the channels’ biophysical properties [5]. α2δ1 was first confirmed by Zhao et al. in liver cancer as a marker for cancer stem cells (CSCs) [6]. The study found that tumor cells with a positive α2δ1 expression had greater tumorigenic capacity *in vitro* and a tendency of chemoresistance. The prognostic value of α2δ1 expression was also evaluated. Subsequently, more and more evidence suggested α2δ1’s prognostic role in patients with lung cancer and ovarian cancer [7,8,9,10]. Inspired by these studies, our laboratory is conducting a series of studies focusing on α2δ1 in head and neck cancer. We demonstrated that α2δ1 positive tumor cells exhibited CSC characteristics in laryngeal cancer [11]. In addition, we are working on α2δ1’s involvement in multi-drug resistance. This article, as a clinical histological evidence, is aimed to determine the value of α2δ1 in clinical practice.

This pilot single-center study is aimed to assess α2δ1 protein expression levels via immunohistochemistry (IHC) in the present study, and identify the reliability of α2δ1 being a prognostic marker in HSCC biopsy specimens collected prior to cancer treatment.

## 2 Materials and methods

### 2.1 Ethics statement

This study was approved by the Bioethics Committee of Beijing Friendship Hospital, Capital Medical University (Batch number: 2019-P2-187-01), and registered in Chinese Clinical Trial Registry (http://www.chictr.org.cn/Registration number: ChiCTR1900027706). All patients understood and signed the informed consent before the experiments. The study conformed with The Code of Ethics of the World Medical Association (Declaration of Helsinki), printed in the British Medical Journal (18 July 1964).

### 2.2 Patients and materials

The eligible HSCC patients were selected based on inclusion and exclusion criteria. Inclusion criteria: HSCC patients with pathological diagnosis; informed consent obtained or waiver of consent; and follow-up information available. Exclusion criteria: Failed to get informed consent; multiple cancers; lack of histological diagnosis; and no follow-up information. Forty-four paraffin-embedded tumor specimens with HSCC were collected from eligible patients who received surgery-oriented comprehensive treatment at Beijing Friendship Hospital, Capital Medical University between May 2014 and January 2019.

For selected patients, two sessions of induction chemotherapy (cisplatin, 75 mg/m^2^, d1; 5-fluorouracil, 750 mg/m^2^), or induction CRT (additional RT dose of 40–50 Gy) were administered as preoperative treatment. The patients would be re-evaluated afterwards, to determine whether to perform open surgery or not. Following the principles of complete tumor removal and functional preservation, surgical procedures were specified based on the workups. Procedures of larynx preservation surgery consisted of partial hypopharyngectomy and partial laryngo-hypopharyngectomy including extended supra-glottic laryngo-hypopharyngectomy, supra-cricoid hemi-laryngo-hypopharyngectomy, and vertical hemi-laryngo-hypopharyngectomy. When larynx could not be preserved, the following procedures could be performed: total laryngectomy with partial hypopharyngectomy and total laryngo-hypopharyngectomy with or without cervical esophagectomy. All patients received adjuvant post-operative radiotherapy delivered by IMRT within 6 weeks: 5 days a week, 2 Gy per day over a 7-week period, reaching a total dose of 70 Gy.

Ten tumor specimens had a pairing peritumoral tissue sample used as the experimental control. All surgeries were performed by the same experienced surgeon. All patients were informed of the purpose of the study and gave informed consent. The age distribution of all patients ranged from 41 to 74 years with a median age of 58.8 ± 7.49 years. Clinicopathological data of the 44 HSCC patients are shown in Table 1. The patients were followed up until September 1st, 2019. The histologic differentiation of HSCC was classified into well, moderately, and poorly differentiated according to the AJCC Cancer Staging Manual, 8th Edition. The definition of OS is the time from the diagnosis of HSCC until death from any cause, while the definition of progression-free survival (PFS) is the time from diagnosis until progression of HSCC or death from any cause.

**Table 1:** Association of α2δ1 expression in hypopharyngeal carcinoma patients with clinicopathological characteristics (Chi-square test)

Parameter	α2δ1 score by IHC			*P*-value
	Total	α2δ1-low	α2δ1-high	
	44	22 (50.0%)	22 (50.0%)	
Age				0.763
<60	23	11 (47.8%)	12 (52.2%)	
≥60	21	11 (52.4%)	10 (47.6%)	
Gender				0.635
Male	39	19 (48.7%)	20 (51.3%)	
Female	5	3 (60.0%)	2 (40.0%)	
Smoking				1.000
Yes	36	18 (50.0%)	18 (50.0%)	
No	8	4 (50.0%)	4 (50.0%)	
Drinking				0.750
Yes	29	14 (48.3%)	15 (51.7%)	
No	15	8 (53.3%)	7 (46.7%)	
Preoperative treatment				0.635
Yes	5	2 (40.0%)	3 (60.0%)	
No	39	20 (51.3%)	19 (48.7%)	
Histological grade				0.082
Well	11	8 (72.7%)	3 (27.3%)	
Poor/moderate	33	14 (42.4%)	19 (57.6%)	
pT stage				0.517
T1–2	14	8 (57.1%)	6 (42.9%)	
T3–4	30	14 (46.7%)	16 (53.3%)	
pN stage				0.365
N0–1	21	9 (42.9%)	12 (57.1%)	
N2–3	23	13 (56.5%)	10 (43.5%)	

### 2.3 IHC

The paraffin-embedded HSCC tissues were cut into 3 μm sections. Then, the sections were baked at 60°C for 2 h, then dewaxed in xylene and rehydrated in graded ethanol solutions. Antigen retrieval was done in citrate buffer in a microwave at 99°C for 15 min. To block the endogenous peroxidase, all sections were incubated with 3% of hydrogen peroxide for 10 min. Blocking was done with 5% of goat serum (#ZLI-9022, ZSGB-BIO, China) for 1 h at ambient temperature. Primary mouse anti-human antibody (CACNA2D1 Monoclonal Antibody [20A], 1:250, # MA3-921, ThermoFisher, USA) incubation was done overnight at 4°C. After washing three times in PBS, the reaction enhancement solution from #PV-9002 kit (ZSGB-BIO, China) was added dropwise for 20 min. Then, the goat anti-mouse secondary antibody from the kit was incubated for 20 min at room temperature. Afterward, diaminobenzidine tetrahydrochloride (#ZLI-9019, ZSGB-BIO, China) was used to develop color for 3 min. Staining with hematoxylin, 0.1% of hydrochloric acid differentiation, dehydration, transparency, and neutral resin sealing were performed successively. Negative control slides omitting the primary antibodies were generated for all assays.

### 2.4 IHC scoring method

Each section was scanned using the Pannoramic 250 Flash Scanner (3DHistech, Budapest, Hungary). All the scanned images were analyzed by Image-Pro Plus software version 6.0 (Media Cybernetics, Inc., Rockville, MD, USA). The magnification was 20× for HE sections and 20×/100× for IHC staining. The positive staining in images was quantified as integral optical density (IOD)/area, that is, mean density = density sum/area sum [12]. In detail, five fields with the strongest expression of α2δ1 were chosen from each section and the average value of mean density was calculated.

### 2.5 Statistical analysis

The patients were separated into two groups (high vs low expression) by median IOD value, 6.035. Chi-square test was used to compare differences between categorical variables. OS and PFS were determined by Kaplan–Meier survival plots and log-rank test. For survival analysis, univariate analysis was conducted using log-rank tests. All variables with *P* < 0.10 on univariate analysis were included in cox regression models. All statistical analyses were achieved by SPSS version 26.0 (IBM Corp, Armonk, NY).

## 3 Results

### 3.1 α2δ1 expression regularly increased in HSCC tissues

**Figure 1:**
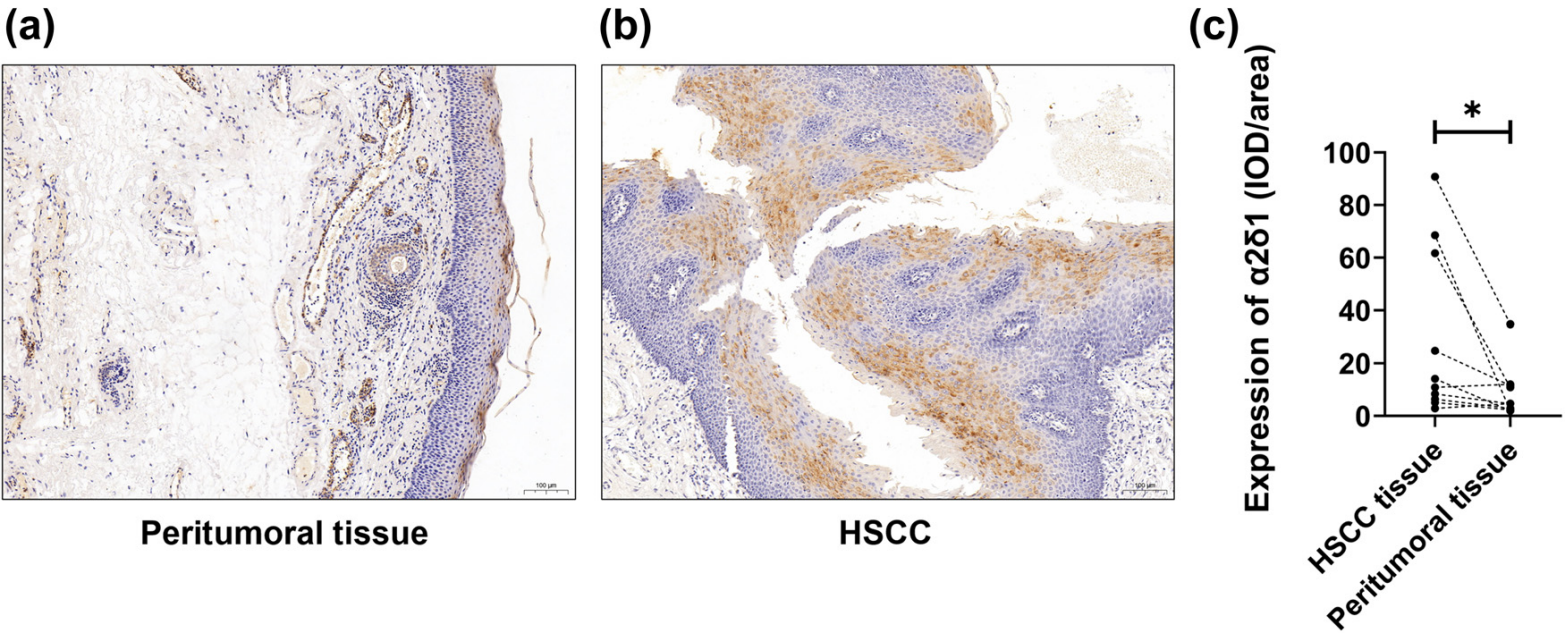
Expression level of α2δ1 in hypopharyngeal squamous cell carcinoma (HSCC) and paired peritumoral mucosal tissues. Expression of α2δ1 in (a) paired peritumoral tissue (100×) and (b) HSCC tissue (100×). (c) Expression level of α2δ1 was significantly increased in paired peritumoral tissue than those in HSCC tissues (*n* = 10, *P* = 0.0359).

**Figure S1:**
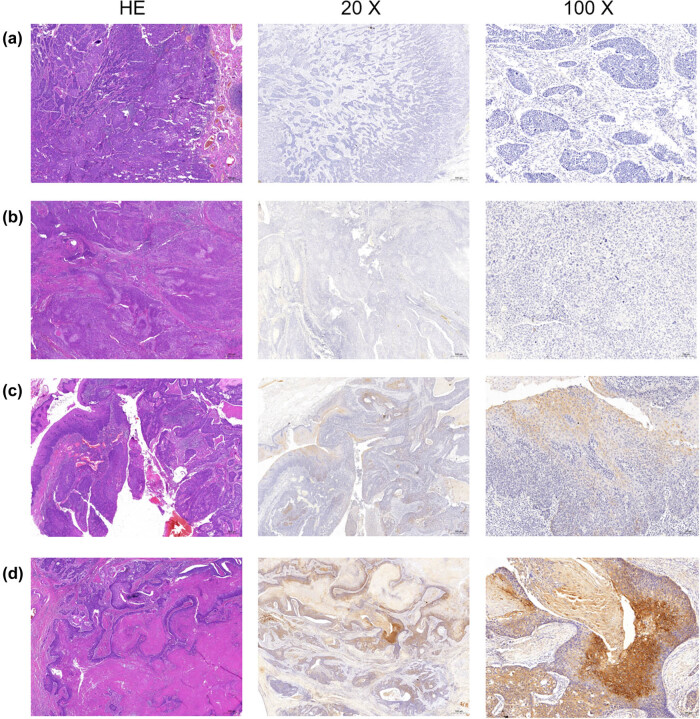
The expression of α2δ1 was variable among HSCC samples. The histological images of 4 representative cases (a–d) were presented. For each case, the HE staining section and immunohistochemistry staining of α2δ1 with different magnifications (20× and 100×) were shown. The expression of α2δ1 varied greatly among these 4 HSCC samples.

According to immunohistochemical analysis, the staining pattern of α2δ1 was principally detected at the membrane and cytoplasm for both HSCC tissues and peritumoral tissues, with yellow/brown particles representing positive expression. The expression of α2δ1 was variable among the 44 HSCC samples (Figure S1). Representative immunohistochemical staining images showed that α2δ1 protein expression significantly increased in HSCC tissues compared to the corresponding peritumoral tissues (Figure 1a and b). Quantitative analyses evaluated in ten paired HSCC and peritumoral tissues confirmed the result of statistical significance (*P* = 0.0359) (Figure 1c).

### 3.2 α2δ1 expression and its clinicopathological associations

Based on the regularly increased expression of α2δ1 protein in HSCC tissues, the clinical value of α2δ1 expression levels was investigated. After calculating the density means of α2δ1, patients were separated into two groups (low vs high expression) according to the median protein expression of tumor tissues. The correlation between α2δ1 expression and clinicopathological characteristics, including age of diagnosis, gender, smoking history, history of alcohol drinking, preoperative treatment, histological grade, pathological T (pT) stage, and pathological N (pN) stage was analyzed. However, no significant association was identified between patients’ clinicopathological characteristics and their α2δ1 protein expression levels (*P* > 0.05; Table 1). The result implied the potential correlation between histological grade (well differentiated vs poorly/moderately differentiated) and α2δ1 protein expression levels (*P* = 0.082). Therefore, we were inclined to hypothesize that the other clinicopathological parameters may not be correlated with cancer cell stemness.

### 3.3 α2δ1 expression was associated with poor OS and PFS

**Figure 2:**
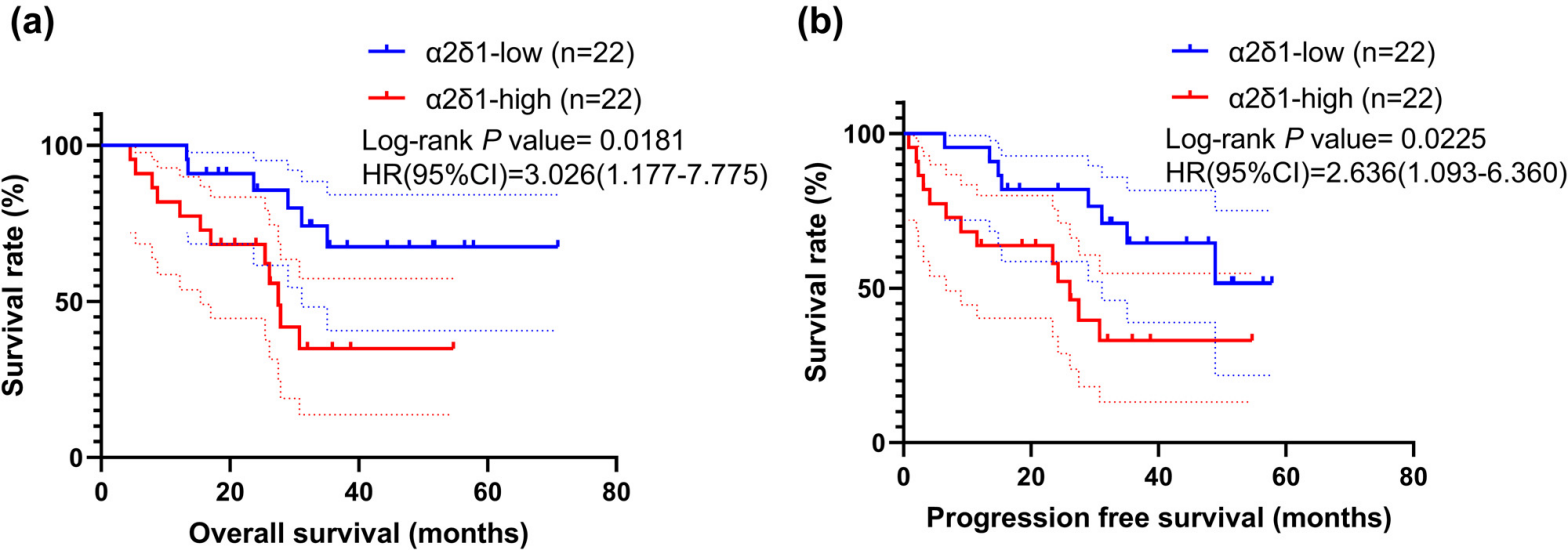
Kaplan–Meier curves for overall survival and progression free survival in HSCC patients (*n* = 44). (a) High α2δ1 expression groups had significantly lower OS than low α2δ1 expression groups. (b) High α2δ1 expression groups had significantly lower PFS than low α2δ1 expression groups. Hazard ratio (HR) and its 95% confidence interval, and Log-rank *P* value are shown.

The OS and PFS of 44 HSCC patients with a positive or negative α2δ1 expression were estimated by Kaplan–Meier statistical analyses. The results showed that the expression of α2δ1 in HSCC was significantly associated with poor OS (*P* = 0.0018, Figure 2a) and PFS (*P* = 0.023, Figure 2b).

### 3.4 α2δ1 expression in HSCC was an independent risk factor for OS and PFS

Univariate and multivariate analyses were tested to estimate the association of α2δ1 protein expression and the above clinicopathological parameters in terms of OS and PFS in HSCC by using cox regression method. Confounding factors were categorized as binary variables: age (<60 vs ≥60), histological grade (well vs poor/moderate), pT stage (T1–2 vs T3–4), and pN stage (N0–1 vs N2–3). The analyses revealed that pT stage and α2δ1 expression were independent risk factors for OS and PFS (*P* < 0.05) when evaluated by multivariate analysis. Patients with α2δ1-high expression had poorer OS and PFS than those with α2δ1-low expression (HR = 4.135 for OS and 4.138 for PFS) (Tables 2 and 3).

**Table 2:** Univariate and multivariate analysis of OS in HSCC (Cox regression)

Covariate	Univariate analysis			Multivariate analysis		
	HR	95% CI	*P*-value	HR	95% CI	*P*-value
Age	0.643	0.244–1.697	0.373			
Gender	0.042	0.000–42.970	0.370			
Smoking	1.284	0.292–5.639	0.741			
Drinking	1.644	0.535–5.046	0.385			
Pre-treat	1.005	0.229–4.406	0.995			
HG	2.675	0.608–11.766	0.193			
pT	3.566	1.009–12.598	0.048	4.231	1.142–15.682	0.031
pN	0.531	0.196–1.443	0.215			
α2δ1	3.530	1.222–10.196	0.020	4.135	1.352–12.643	0.013

HG: histological grade; Pre-treat: preoperative treatment.

**Table 3:** Univariate and multivariate analysis of PFS in HSCC (Cox regression)

Covariate	Univariate analysis			Multivariate analysis		
	HR	95% CI	*P*-value	HR	95% CI	*P*-value
Age	0.680	0.258–1.790	0.435			
Gender	0.041	0.000–28.893	0.340			
Smoking	1.510	0.343–6.640	0.586			
Drinking	1.799	0.586–5.528	0.305			
Pre-treat	0.897	0.205–3.925	0.885			
HG	2.953	0.673–12.966	0.151			
pT	3.388	0.963–11.920	0.057	3.991	1.092–14.580	0.036
pN	0.543	0.200–1.472	0.230			
α2δ1	3.592	1.250–10.324	0.018	4.138	1.382–12.389	0.011

HG: histological grade; Pre-treat: preoperative treatment.

## 4 Discussion

α2δ1 dysregulation has been found in multiple cancer types. As far as we know, this study is the first to explore the protein expression level and prognostic significance in HSCC. We wish that our findings will make contributions to the diagnosis and treatment of the disease and improve the prognostic expectations for HSCC patients.

In our study, we first analyzed the expression status of α2δ1 in paired HSCC and normal hypopharyngeal mucosa tissues by IHC. α2δ1 expression yielded the highest staining at the tumor center. Our results proved that α2δ1 protein expression was elevated in HSCC tissues compared with the matched peritumoral hypopharyngeal tissues. Then, the prognostic significance of α2δ1 in HSCC tissue was studied. From the Kaplan–Meier survival curves of our cohort, patients’ α2δ1 protein expression was observed to be correlated with their clinical outcomes. Also, the patients with higher expression level of α2δ1 were inclined to have both shorter OS and PFS statistically. The multivariate cox regression analysis revealed α2δ1 and pT acted as independent predictors for OS in HSCC patients.

It is widely acknowledged for oncologists that higher pT staging indicates worse OS and PFS in HSCC, so the result of pT being an independent risk factor for survival is not surprising. However, the clinical significance of α2δ1 expression in HSCC has not been reported. The result that the expression of α2δ1 has the parallel hazard ratio with pT as independent predictors for OS and PFS is inspiring. It suggests that patients with high α2δ1 are supposed to be treated with more aggressive strategies and close follow-ups. Considering the fact that α2δ1 was reported as a functional marker for CSCs [8,10,11], it might serve as a potential candidate for therapeutic target of HSCC in the future.

The clinical histological analysis of α2δ1 has been conducted in liver cancer and epithelial ovarian cancers [6,9]. Both the studies confirmed higher α2δ1 protein expression indicating worse clinical outcome, which was similar to our study. In addition, the former study also found higher α2δ1 protein expression in tumor tissues than in peritumoral tissues. And the latter study found that α2δ1 protein expression of tumor was correlated with its histological grade, though we did not reach this conclusion.

The mechanism underlying the prognostic value of α2δ1 was mainly because of its role as a marker for CSCs. α2δ1 subunit participated in the formulation of T-type and L-type voltage gated calcium channel (VGCC). It was reported that T-type VGCC was involved in the biological procedure of cell cycle progression and stemness maintenance [13], while L-type VGCC was associated with stem cell differentiation [14,15]. Intracellular calcium played an essential role in stem cells’ self-renewing, proliferation, and differentiation [14,16]. Further experiments confirmed α2δ1-positive tumor cells possessed the capacity of tumorigenic *in vitro* and *in vivo*, chemoresistance, and radioresistance [6,7,8], providing more evidence to its role as a CSC marker and a prognostic marker of cancer. However, no significant correlation was found between the α2δ1-positive staining and any other clinicopathologic factor other than histological grades, OS, and PFS in our study. Hence, we would like to deduce that the other clinicopathological characteristics may not associate with cancer cell stemness or chemo-radioresistance.

We tried to mine the mRNA transcriptional and survival data of CACNA2D1 from online databases such as the cancer genome atlas and GEO datasets, in order to provide more evidence for the prognostic value of α2δ1 according to the bioinformatic methods we used before [17]. But the mRNA level of CACNA2D1 was not significantly altered between tumor and normal tissues, and did not correlate with the patients’ survival time as well (data not shown). The discrepancy might come from the two techniques, RNA-seq and IHC, based separately on RNA and protein level. We deemed that IHC straightforwardly reflecting the protein expression level, and as the most widely used method in clinical practices, was convincing.

IHC has gained significance in diagnosis of diseases and has already taken a crucial position in predicting patients’ prognosis [18]. The result of IHC is usually interpreted by visual assessment. However, the subjectivity and interpretation reproducibility of IHC could be inevitable pitfalls [19]. New demands on the accuracy, reproducibility, and quality of IHC are required for medicine in the new era [20]. To meet these criteria, Image-Pro Plus software was employed to quantify IHC staining levels automatically, and the results were satisfactory.

HSCC being a relatively uncommon disease, only 44 patients were enrolled in our study. Although the study design was elaborated, the limited number of patients and the nature of retrospective study made potential selection bias and confounding bias inevitable. To further guarantee α2δ1’s predictive role in the prognosis of HSCC patients, a multicenter, prospective study with more participants was needed.

## 5 Conclusion

This study distinguishes itself from the others by first exploring the expression levels of α2δ1 in HSCC tumor and adjacent normal tissues and by showing the prognostic significance of α2δ1 in HSCC tissues. The present study suggested that α2δ1 could serve as a new prognostic biomarker for HSCC based on regular IHC analysis.

## Abbreviations

α2δ1: voltage-dependent calcium channel subunit alpha-2/delta-1
CSCs: cancer stem cells
HSCC: hypopharyngeal squamous cell carcinoma
IHC: immunohistochemistry
IOD: integral optical density
OS: overall survival
PFS: progression-free survival
VGCC: voltage gated calcium channel
